# Characterization of Membrane Lipidome Changes in *Pseudomonas aeruginosa* during Biofilm Growth on Glass Wool

**DOI:** 10.1371/journal.pone.0108478

**Published:** 2014-09-29

**Authors:** Hayette Benamara, Christophe Rihouey, Imen Abbes, Mohamed Amine Ben Mlouka, Julie Hardouin, Thierry Jouenne, Stéphane Alexandre

**Affiliations:** 1 Polymères Biopolymères, Surfaces Laboratory - UMR 6270, CNRS - Université de Rouen, Mont-Saint-Aignan, France; 2 Normandie Université, Mont-Saint-Aignan, France; 3 PISSARO proteomic facility, IRIB - Université de Rouen, Mont-Saint-Aignan, France; Centre National de la Recherche Scientifique, Aix-Marseille Université, France

## Abstract

Bacteria cells within biofilms are physiologically distinct from their planktonic counterparts. In particular they are more resistant to detrimental environmental conditions. In this study, we monitored the evolution of the phospholipid composition of the inner and outer membranes of *P. aeruginosa* during the biofilm formation (i.e., from 1-, 2-, to 6-day-old biofilm). Lipidome analyses were performed by electrospray ionization mass spectrometry. In addition to the lipidomic analysis, the fatty acid composition was analysed by gas chromatography/mass spectrometry. We found that the lipidome alterations of the inner and the outer membranes varied with the biofilm age. These alterations in phospholipid compositions reflect a higher diversity in sessile organisms than in planktonic counterparts. The diversity is characterized by the presence of PE 30∶1, PE 31∶0 and PG 31∶0 for the lower masses as well as PE 38∶1, 38∶2, 39∶1, 39∶2 and PG 38∶0, 38∶1, 38∶2, 39∶1, 39∶2 for the higher masses. However, this lipidomic feature tends to disappear with the biofilm age, in particular the high mass phospholipids tend to disappear. The amount of branched chains phospholipids mainly located in the outer membrane decreased with the biofilm age, whereas the proportion of cyclopropylated phospholipids increased in both membranes. In bacteria present in oldest biofilms, i.e., 6-day-old, the phospholipid distribution moved closer to that of planktonic bacteria.

## Introduction

*Pseudomonas aeruginosa* is well known as an opportunistic pathogen that causes a variety of diseases in individuals predisposed to infections as the result of severe burns, wounds, urinary tract or corneal injury, or immunocompromised status [1] and is the leading cause of mortality and morbidity in cystic fibrosis (CF) patients [2]. This bacterium is characterised by its innate resistance to antibiotics due to a low outer membrane (OM) permeability and the presence of active efflux (Mex) systems [3], [4].

*P. aeruginosa* can form biofilms which can be defined as biopolymer matrix-enclosed microbial populations adhering to each other and/or surfaces [5]. *P. aeruginosa* biofilms are involved in the pathogenesis of urinary, ventilator-associated pneumonia, peritoneal dialysis catheter infections, bacterial keratitis, otitis externa, burn wound infections and chronic bronchitis in CF patients [5]. Bacterial biofilm infections are particularly problematic because sessile bacteria are drastically more resistant to antimicrobials as compared with planktonic counterparts [6], [7].

The protective mechanisms involved in biofilms resistance appear to be distinct from those that are responsible for conventional antibiotic resistance and it is becoming evident that biofilm resistance is multifactorial [8]. Poor antibiotic penetration, nutrient starvation, slow growth, adaptive stress responses and formation of persister cells are hypothesized to constitute a multi-layered defence [9]. The biofilm phenotype of *P. aeruginosa* appears regulated more at the translational and perhaps post-translational levels than at the transcriptional level, as highlighted by the discrepancies between transcriptomics [10], [11] and proteomics [12], [13].

Due to the importance of biofilms in industry, environment and for human health, the physiology of sessile micro-organisms has been widely investigated in the last decade. Initially, genomics investigations were performed by screening of biofilm defective mutants [14]. Then, transcriptomics [15] and proteomics [13] approaches were used to identify genes or proteins up- or down-regulated in sessile bacteria. More recently, metabolomics investigations were initiated to characterize the various metabolic states within biofilms [16].

Although, it is well known that inner membrane (IM) is a central component for microorganism survival, which are not insulated from extrinsic physical and chemical factors [17], few works were surprisingly devoted to lipidomics of biofilm organisms up to now. Abdul Lattif et *al* [18] compared the lipid profiles of biofilm and planktonic *Candida albicans* cells. They showed that significant differences existed in lipid composition according to the growth mode and the developmental phases. In a previous work, we pointed out a drastic decrease of the uneven chain phospholipids and an accumulation of long chain lipids in sessile *P. aeuginosa* cells as compared with planktonic counterparts, suggesting a higher lipid stability in the bilayer and a decrease in membrane fluidity [19].

The objective of the present study was to monitor the distribution of lipid classes during the biofilm growth (i.e. 1-, 2- to 6-day-old biofilms). Phospholipids quantification in *P. aeruginosa* IM enriched samples and OM enriched samples confirmed differences between biofilms and planktonic bacterial lipidomes but also demonstrated a biofilm age-dependence of the lipidomic alterations.

## Materials and Methods

### Bacterial strain and preculture

*P. aeruginosa* PAO1 strain was used. Bacteria were stocked in 30% (v/v) glycerol. Preculture was performed in a 50 mL flask containing 1 mL of bacteria stock suspensions and 10 mL of Muller-Hinton broth (MHB, Difco). The flask was incubated at 37°C on a rotary shaker (140 rpm) for 18 h.

### Planktonic and biofilm cultures

For planktonic cultures, a preculture was used to inoculate (final concentration, 10^7^ Colony Forming Units (CFU)/ml) 100 mL of Minimal Glucose Medium (MGM) of the following composition: 15 g/L Tris-HCl, 0.6 g/L Tris-base, 0.5 g/L NH_4_Cl, 2 g/L yeast extract, 0.05 g/L CaCl_2_, 0.05 g/L MgSO_4_, 0.005 g/L FeSO_4_, 0.005 g/L MnSO_4_, 15 g/L glucose. Cultures were incubated on a rotary shaker (140 rpm) at 37°C for 24 h to reach the stationary phase of growth.

For biofilm cultures, 100 mL of MGM containing 2 g (total area 2800 cm^2^) of sterile glass wool were inoculated at 10^7 ^CFU/mL from a pre-culture as previously described [12], [19]. Biofilms were grown under slight agitation (20 rpm) at 37°C for 1, 2 or 6 days. The clear formation of a biofilm on glass wool has been checked by scanning electron microscopy. Support samples were taken after incubation for 1 day and rinsed twice with sterile phosphate buffer. Samples were fixed in a 2% glutaraldehyde, 0.1 M cacodylate buffer (pH 7.4) for 30 min and rinsed (3×10 min) in 0.2 M cacodylate buffer (pH 7.4). Samples were then dehydrated by passing them through the following ethanol series: 30%, 50%, 80%, each for 10 min; 100% ethanol, 2 10 min. Support samples were then dried at 37°C for 24 h. Once coated with gold - palladium (Sputtering Device), samples were examined using scanning electron microscopy (Cambridge S200).

### Bacteria recovery

After 24 h of incubation, planktonic organisms were recovered by centrifugation (10 min at 2600×g at 4°C). Sessile bacteria were recovered as described previously [19]. Briefly, glass wool was aseptically removed after 1, 2 or 6 days of incubation, and washed twice in 0.1 M, pH 7 Phosphate Buffer Saline (PBS) to release weakly attached cells. It was then placed in sterile flasks containing 30 g of glass beads (diameter, 3 mm) and 50 mL of PBS. Bacteria were released from the substratum by vigorous shaking for 20 min. They were then harvested by centrifugation ((10 min at 2600×g) at 4°C) and resuspended in 5 ml of sterile Milli-Q water.

### Membrane extractions

Bacterial IM enriched samples extraction was carried out following the spheroplast protocol first described by Mizuno and Kageyama [20]. After centrifugation (see above) bacterial pellets were washed in 10 mL of 20% (w/v) sucrose. After another centrifugation, pellets were weighted and resuspended in a digestion solution of the following composition: for 1.5 g bacteria wet weight, 18 mL of 20% sucrose, 9 mL of 2 M saccharose, 10 mL of 0.1 M Tris-HCl, 0.8 mL of 1% EDTA, 1.8 mL of 1% lysozyme, 1 µL of 1 mg/mL RNase and 5 µL of 20 mg/mL DNase. The solution was incubated at 37°C. Spheroplast formation was monitored by optical microscopy. When only ovoid forms were observed, the suspension was centrifuged at 30°C for 15 min at 5200×g to recover spheroplasts. The pellet was resuspended in 5 mL of 0.01 M PBS and was subjected to sonication (cycles of 30 s for 2 min). The suspension was then centrifuged at 30°C, for 20 min at 5200×g. The supernatant, containing IM, was diluted in 100 mM sodium carbonate, stirred at 4°C for 1 h to separate soluble and insoluble phase. Then it was ultracentrifuged (60,000×g for 1 h at 4°C) to harvest IM enriched samples. Pellets were washed twice with 40 mM Tris buffer (pH 7) and freezed at −20°C.

In order to harvest OM enriched samples, the digestion time was divided by a factor of 2. The suspension was then centrifuged at 30°C for 15 min at 5200×g and the supernatant containing mostly OM was collected and freezed at −20°C.

### Outer membrane proteins detection in membrane extracts

In order to detect the cross-contamination in the different membrane extracts, OM proteins (OMP) fractions were identified using nanoLC-MS/MS. Proteins were digested according to the following procedure. Twenty-five µg of proteins were alkylated with 25 mM iodoacetamide for 45 min in the dark. The protein sample was mixed with SDS loading buffer (63 mM Tris-HCl, pH 6.8, 10 mM DTT, 2% SDS, 0.02% bromophenol blue, 10% glycerol), then loaded onto a SDS-PAGE stacking gel (7%). A short electrophoresis was performed (10 mA, 15 min). After migration, the gel were stained with Coomassie blue and destained with a solution containing 50% ethanol, 10% acetic acid and 40% deionized water. The revealed protein band was excised, washed with water, submitted to protein digestion with trypsin (0.5 µg per band). The digestion was achieved after 3 h at 37°C. Several steps of peptide extraction were performed in H_2_O/ACN/TFA (49.5/49.5/1). The peptides were dried and stored at −20°C.

The nanoLC-MS/MS analysis of the eluates was repeated twice. All experiments were performed on a LTQ-Orbitrap Elite coupled to an Easy nLC II system (both from Thermo Scientific). Samples were injected onto an enrichment column (C_18_ PepMap100, Thermo Scientific). The separation was achieved with an analytical column needle (NTCC-360/100-5-153, NikkyoTechnos). The mobile phase consisted of H_2_O/FA 0.1% (buffer A) and ACN/FA 0.1% (buffer B). Tryptic peptides were eluted at a flow rate of 300 nL/min, using a three-step linear gradient: from 2 to 40% B over 105 min, from 40 to 80% B in 4 min and at 80% B for 11 min.

The mass spectrometer was operated in positive ionization mode with a capillary voltage and a source temperature set at 1.6 kV and 275°C, respectively. The samples were analyzed using the collision induced dissociation. The first scan (MS spectra) was recorded in the Orbitrap analyzer (R = 60,000) with the mass range m/z 400–1800. Then, the 20 most intense ions were selected for MS^2^ experiments. Singly charged species were excluded for MS(n) analysis. Dynamic exclusion of already fragmented precursor ions was applied for 30 s, with a repeat count of 1, a repeat duration of 30 s and an exclusion mass width of ±5 ppm. Fragmentation occurred in the linear ion trap analyzer with collision energy of 35. All measurements in the Orbitrap analyzer were performed with on-the-fly internal recalibration (lock mass) at m/z 445.12002 (polydimethylcyclosiloxane).

Raw data files were then processed using Proteome Discoverer 1.3 software (Thermo Scientific). Peak lists were searched using the MASCOT search engine (Matrix Science) against the *P. aeruginosa* PA01 database (http://www.pseudomonas.com) [21]. Database searches were performed with the following parameters: 2 missed cleavage sites allowed; variable modifications: carbamidomethylation of cystein, and oxidation of methionine. The parent ion and daughter ion tolerances were 10 ppm and 0.5 Da, respectively. Subcellular localizations were predicted by PSORTb V3.0.

IM and OM enriched membrane extracts from 3 independent bacterial cultures were analysed. In order to compare the results from the IM and OM enriched membrane extracts, peptide spectral match (PSM) values for the porin and structural outer membrane porin OprF precursor (PA14_41570) were used. The mean PSM ratio was calculated and the result is given with an error corresponding to a 95% confidence interval.

### Inner membrane proteins detection in membrane extracts

To evaluate the proportion of IM proteins in the membrane extracts, NADH oxidase assays were performed, NADH oxidase being described as an IM marker [22]. The overall protein concentration in the samples was measured using Bradford methods. Then, membrane samples containing 50 or 100 µg total protein was added in a mixture containing 50 mM Tris pH 7.5, 0.2 mM dithiothreitol, 0.12 mM NADH, in a volume of 3 ml. The decrease in absorbance of NADH at 340 nm was followed at room temperature in a Varian Cary 100Bio spectrophotometer for 30 min. Three different extracts obtained from 3 independent bacterial cultures were tested using 2 concentrations of total proteins per extract (16.6 and 33.3 µg/mL). The initial rates of NADH oxidation were measured and their mean values per µg of proteins were calculated. The mean ratio of the average rates between the OM enriched samples and the IM enriched samples was calculated (with an error corresponding to a 95% confidence interval).

### Lipid extractions

Lipid extraction was carried out according to Bligh and Dyer protocol [23]. For 1 mL of membrane extract, 3.75 mL of chloroform: methanol (1∶2 v/v) solution was added. The mixture was sonicated for 5 min and vortexed for 15 min until obtaining a milky-mixture. After adding 1.25 mL of chloroform, the mixture was vortexed for 1 min. A volume of 1.25 mL of 1 M NaCl aqueous solution was added and the mixture vortexed again for 15 min. Finally, the mixture was centrifuged (670×g for 10 min at 30°C) to separate organic and aqueous phase. The organic phase was recovered with a Pasteur pipette and 1.88 mL of chloroform was added to the aqueous phase. After stirring for 15 min, the mixture was centrifuged (670×g for 10 min at 30°C) to separate the two phases. The aqueous phase was removed and organic phases were mixed and evaporated under argon.

To degrade and eliminate proteolipids, 1 mL of methanol was added to lipid extracts. The mixture was vortexed for 5 min. Methanol was then evaporated. A chloroform: methanol (1∶2 v/v) solution was added and the mixture was centrifuged (670×g for 10 min at 30°C), to sediment proteins. The organic phase was recovered and evaporated under argon. The lipid extracts were conserved at −20°C.

### Mass spectrometry analyses of phospholipid extracts

Lipid extracts were analysed by Electrospray Ionization Mass Spectroscopy (ESI-MS) as previously described [19]. A Linear Quadruple Ion Trap (QTRAP, AB Sciex Instruments) spectrometer, equipped with a turbo spray ionization source heated to 300°C, was used. The potential applied during the acquisition was – 4500 V. The mass calibration and resolution were made according to the procedure of manufacturer specifications.

The concentrations of the lipid solutions were checked by the method described by Raheja et *al* [24] and by measuring the surface pressure of Langmuir film for each lipid extract [19]. Lipid extracts were diluted in a chloroform: methanol (80∶20 v/v) solution. The volume of solvent was adjusted in order to obtain a 1 µM solution. The solution was subjected to sonication for 1 h to obtain stable and homogeneous solution. Samples were infused at 10 µL/min flow rate in negative ionization mode. Spectra were acquired at 1000 amu.sec^−1^ over the 50–1700 m/z range.

Phospholipid spectra were analysed via a home-made python script under the scientific data analysis software SciDaVis (http://scidavis.sourceforge.net/). Three IM enriched samples lipid extracts and two OM enriched samples lipid extracts were used for each culture condition. For each extracts, three spectra were analysed and all these results were combined. The mean value were calculated and the results are presented with error bars corresponding to a 95% confidence interval. Comparisons with the 3 biofilm growth conditions were analysed using one-way analysis of variance (ANOVA). In addition an ANOVA test including the planktonic mode of growth was performed. The ANOVA was performed using the script from Pr. H. Arsham (http://home.ubalt.edu/ntsbarsh/Business-stat/otherapplets/pvalues.htm).

### Bacterial total fatty acid extraction

The fatty acid extraction was performed as described by Steger et *al* [25] and Hoffmann et *al* [26]. Planktonic and biofilm bacteria were harvested at 2600×g for 10 min at 4°C. The resulting pellets were resuspended into 2 mL Milli-Q water and then lyophilised for 2 days at −62°C. Five to 10 mg of lyophilised bacteria were used in each extraction. For the saponification reaction, 1 mL of sodium hydroxide (3.75 M) in 1∶1 methanol-water was first used at 80°C for 30 min to release fatty acids. The second step, i.e., the transesterification (fatty acids methylation) was performed with 2 mL of 10% hydrochloric acid in methanol at 80°C for 10 min. This second step allows the decrease of fatty acid polarity and to obtain stable molecules. In the last step, fatty acids were separated using 1.5 mL of 1∶1 hexane-methyl tert-butyl ether mixture. Organic phases containing fatty acids were then washed with 3 mL of 0.3 M sodium hydroxide, and submitted to an additional extraction with 1 mL of hexane. Fatty acid methyl esters (FAMEs) were thus obtained.

### Fatty acids analysis by Gas Chromatography-Mass Spectrometry (GC-MS)

FAMEs were analysed by GC-MS. A capillary column (30 m×0.25 µm×250 µm id) was used to separate fatty acids. Injection temperature was 250°C. Oven temperature ranged from 60 to 300°C at 8°C/min rate. One hundred microlitres of cyclohexane were added to 100 µL of samples. MgSO_4_ was then added to obtain dry samples. A volume of 0.5 µL of the mixture was injected with splitless injector flow. Resulting chromatogram picks were then identified by mass spectrometry.

## Results and Discussion

### Biofilm formation

**Figure 1:**
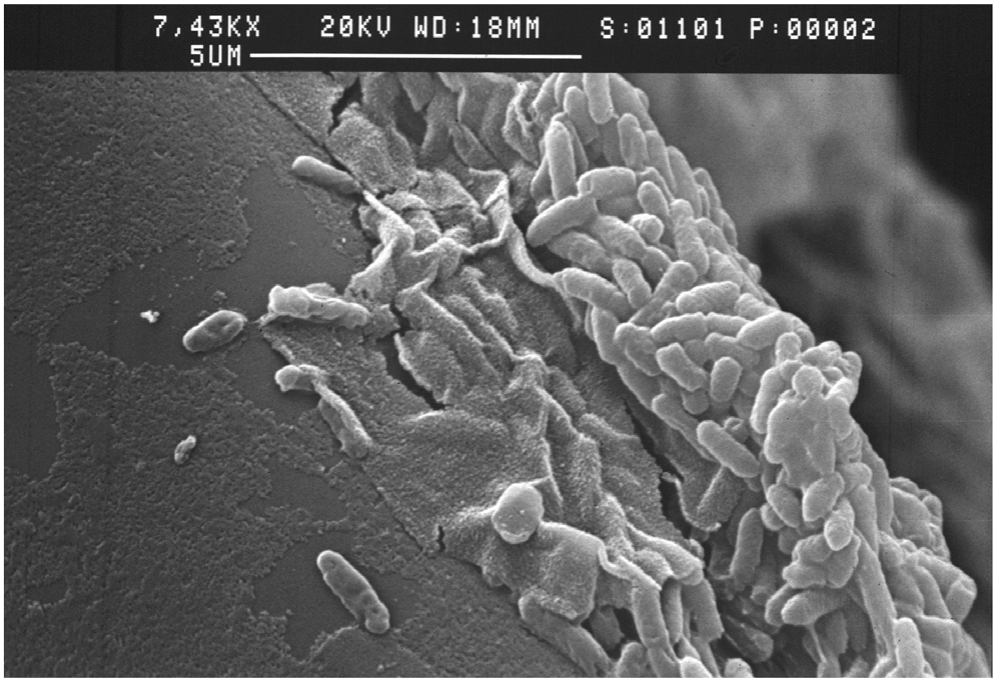
Formation of *P. aeruginosa* biofilms on glass wool after 24 h as observed by scanning electron microscopy. For complementary information on *P. aeruginosa* biofilms grown on glass wool, see [27].

After one day, *P. aeruginosa* formed a biofilm as shown on **figure 1**. Individual bacteria were still observed, but a biofilm is clearly already formed. We previously showed that biofilms cultured in this manner produce an extensive canopy after two days [27].

### Fatty acids profiles in planktonic and biofilm *P. aeruginosa* cells

*P. aeruginosa* lipids were saponified in order to get the fatty acids composition in planktonic and biofilm bacteria at various times of biofilm formation (**Table 1**). The list of fatty acids accords well with that previously published for sessile *P. aeruginosa* cells [28]. The notable exceptions are 16-CH_3_-C17∶0, 17-CH_3_-C18∶0 and C20∶0, which were here observed whereas previously absent [28]. However, we found those fatty acid in only 1-day-old biofilm organisms. The study by Chao et al [28] was conducted using 2-day-old biofilms, and this may explain this discrepancy. This suggests that the diversity in fatty acid composition is higher in young biofilm bacteria than in older (e.g., 6-day-old) ones.

**Table 1:** Fatty acids profiles in planktonic and biofilm *P. aeruginosa* cells.

Fatty acid	retentiontime	mass (methyl esterfatty acid)	Planktonic (%)	1 day-old biofilm(%)	2 days-old biofilm(%)	6 days-old biofilm(%)
3-OH-C10∶0	13∶15	202	2.6	0.0	0.0	0.0
C12∶0	14∶11	214	2.7	0.3	0.0	0.2
2-OH-C12∶0	15∶53	230	1.2	0.2	0.0	0.0
3-OH-C12∶0	16∶16	230	2.0	2.8	0.4	3.6
12-CH3-C13∶0	16∶34	242	0.0	1.7	0.4	0.0
C14∶0	17∶02	242	1.3	2.3	0.2	0.9
13-CH3-C14∶0	17∶59	256	0.7	1.4	0.4	0.0
12-CH3-C14∶0	18∶03	256	0.0	21.0	6.3	0.0
C15∶0	18∶22	256	0.0	1.5	0.4	0.3
14-CH3-C15∶0	19∶12	270	0.0	2.3	1.2	0.2
C16∶1	19∶25	268	2.6	2.8	12.3	6.3
C16∶0	19∶42	270	64.9	28.4	39.5	45.0
15-CH3-C16∶0	20∶27	284	0.0	1.1	0.0	0.0
14-CH3-C16∶0	20∶34	284	0.0	5.1	0.4	0.0
C17∶0cyc(9,10)	20∶45	282	3.2	0.9	2.0	2.7
C17∶0	20∶53	284	0.0	1.9	1.9	0.1
16-CH3-C17∶0	21∶39	298	0.0	2.8	0.2	0.2
C18∶1	21∶51	296	13.0	15.1	30.0	34.2
C18∶0	22∶04	298	0.7	6.3	2.37	0.2
17-CH3-C18∶0	22∶53	312	0.0	2.0	0.16	0.1
C19∶0cyc(11,12)	23∶04	310	6.5	0.9	4.0	6.3
C20∶0	24∶18	326	0.0	2.8	0.1	0.0
**Linear saturated fatty acids**			**69.5**	**40.0**	**42.2**	**46.4**
**Linear unsaturated fatty acids**			**15.6**	**17.9**	**42.3**	**40.5**
**Cyclic fatty acids**			**9.7**	**1.7**	**5.9**	**9.0**
**Branched chain fatty acids**			**0.7**	**37.5**	**9.1**	**0.6**
**Hydroxy fatty acids**			**4.5**	**2.8**	**0.4**	**3.6**

In most samples, the two main fatty acid constituents were the saturated fatty acid C16∶0 and the unsaturated fatty acid C18∶1, in accordance with observations by Chao et *al* [28]. The ratio between C16∶0 and C18∶1, we observed, was around 5 in planktonic cells but decreased to 1.9 in 1-day-old and to 1.3 in 6-day-old biofilm cells. This is in contradiction with previous data [28] showing a mean ratio of 0.5 whatever the sample. Another discrepancy came from branched chains and hydroxy fatty acids. Thus, for 2-days-old biofilms, branched chains fatty acids represent here 9.1% of the total fatty acids while they were previously undetected [28]. It should also be noticed that for 1-day-old biofilm cells, the anteiso methylated-C14∶0 itself represented 21% of the total fatty acids. In contrary in 2-days-old biofilms, hydroxy fatty acids proportion was here 0.4% while it was 19.9% in the previous investigation [28]. However in both studies, the proportion of cyclopropylated fatty acids increased during the biofilm formation, reaching in 6-day-old biofilm cells, the amount observed in planktonic counterparts.

These results illustrate the experimental difficulties in fatty acid analysis. Another difficulty is in the interpretation of the results. Indeed, while some fatty acids came from phospholipids hydrolysis, others may have lipopolysaccharide, and/or lipoproteins, as origin. We then monitored the evolution of the total phospholipids in the inner and outer membrane of the sessile bacteria. The main advantage of such approach is that lipid extracts are used for the ESI-MS analysis [29]. This procedure is simple and do not need further chemical modifications (e.g., hydrolysis) or extraction. Moreover, the amounts of lipid required is low. The risk of contamination or error analysis is consequently limited.

### Evolution of phospholipid distribution during biofilm formation

The phospholipid composition of IM and OM enriched samples were monitored over a 1 to 6 days incubation period and compared with that of planktonic bacteria. Since it is difficult to fully separate OM from IM we evaluated the cross-contamination in the samples. Membrane samples were analysed with nanoLC-MS/MS. It clearly showed a higher proportion of OMPs in the OM enriched samples than in the IM enriched ones as expected. Among these OMPs, we used the major porin and structural outer membrane porin OprF precursor (PA14_41570) to evaluate the cross-contamination within the two samples as already described [30]. The ratio of PA14_41570 amount between the OM and IM enriched samples was 1.7±0.3.

In the case of the IM protein samples, due to the sample complexity and the low expression level of each protein, we evaluated the cross-contamination by the measure of the NADH oxidase activity [22], [30]. Actually we observed NADH oxidase activity in both samples. However the enzymatic activity in the OM enriched samples was twice lower than in IM enriched samples. The oxidase activity ratio between the OM enriched samples and the IM enriched samples was 0.5±0.1.

These results demonstrate the efficacy of the membrane fragments enrichment. It shows that the proportion of IM is 2 times higher in the IM enriched sample compared to OM enriched samples and that the proportion of OM is 1.7 times higher in the OM enriched sample compared to IM enriched samples.

Phosphatidylglycerol (PG) and phosphatidylethalonamine (PE) were the most detected phospholipids. Phosphatidic acid, cardiolipin and ornithine lipids (OL) were also detected. However the peak intensity for these phospholipids were very low and to observe any significant changes. Then, We consequently focused on the changes in PGs and PEs amounts (**table 2**). In the text and table 2, phospholipids are identified as C:U with C being the total number of carbon atoms in the aliphatic chains and U being the equivalent number of unsaturation. The equivalent number of unsaturation accounts for either the presence of unsaturation or the presence of a cyclopropyl group.

**Table 2:** Distribution of the predominant inner and outer membrane phosphatidylethanolamines and phosphatidylglycerols in planktonic and biofilms *Pseudomonas aeruginosa* (results are given with 95% confidence interval).

			PEs					PGs				
PL*	R	R’	mass	plankt	BF 1 day	BF 2 days	BF 6 days	mass	plankt	BF 1 day	BF 2 days	BF 6 days
32∶1	C16∶0	C16∶1	688.49	4.8±0.6	6.6±0.5	6.6±0.3	6.0±0.5	719.49	4.4±2.0	4.7±0.8	4.8±0.9	4.9±1.1
	C14∶0	C18∶1		*5.4±0.6*	*5.6±0.5*	*7.3±0.5*	*7.3±0.5*		*n.d.*	*n.d.*	*n.d.*	*n.d.*
	CH3-C14∶0[traces]	C17∶0cyc [traces]										
**32∶0**	**C16∶0**	**C16∶0**	**690.51**	**8.6±0.6**	**8.2±0.5**	**11.0±0.3**	**8.8±0.5**	**721.50**	**5.6±2.0**	**7.2±0.8**	**4.4±0.9**	**4.4±1.1.**
	**C14∶0**	**C18∶0**		***7.0±0.5***	***5.4±0.5***	***13.6±0.5***	***5.6±0.5***		***n.d.***	***n.d.***	***n.d.***	***n.d.***
33∶1	C16∶0	C17∶0cyc(9,10)	702.51	5.9±0.4	3.5±0.3	4.6±0.3	14.5±0.7	733.50	6.2±0.8	6.0±0.5	7.6±0.5	12.9±0.5
	CH3-C16∶0	C16∶1		*5.9±0.5*	*4.6±0.5*	*2.6±0.3*	*21.1±0.9*		*9.5±0.8*	*5.9±0.5*	*5.7±0.5*	*2.1±0.5*
**33∶0**	**C16∶0**	**CH3-C16∶0**	**704.52**					**735.52**	**2.7±0.8**	**4.2±0.5**	**2.5±0.5**	**4.1±0.5**
	**CH3-C14∶0**	**C18∶0**		***2.6±0.8***	***2.5±0.7***	***2.1±0.5***	***2.6±0.6***		***8.2±0.5***	***2.2±0.7***	***3.0±0.5***	***3.1±0.6***
34∶2	C16∶1	C18∶1	714.51	5.2±1.6	13.7±0.7	7.7±0.9	8.2±1.0	745.50	2.4±0.5	7.4±0.5	3.5±0.5	5.3±0.5
				*6.2±0.8*	*11.7±0.9*	*8.2±0.9*	*6.5±0.9*		*4.1±0.8*	*7.1±0.6*	*8.0±0.6*	*10.0±0.6*
**34∶1**	**C16∶0**	**C18∶1**	**716.52**	**44.8±1.8**	**35.3±0.7**	**35.4±0.9**	**30.7±1.0**	**747.52**	**21.9±0.5**	**17.1±0.5**	**17.9±0.5**	**19.5±0.5**
	**C16∶1**	**C18∶0**		***45.8±1.0***	***31.4±0.9***	***45.2±0.9***	***26.9±0.8***		***25.9±0.8***	***23.4±0.7***	***33.4±0.7***	***28.2±0.8***
	**CH3-C14∶0** **[traces]**	**C19∶0cyc [traces]**										
35∶2	C16∶1	C19∶0cyc(11, 12)	728.52	4.2±0.8	4.0±0.5	4.6±0.5	4.6±0.5	759.59	n.d.	6.9±0.5	4.9±0.5	n.d.
	C17∶0cyc(9,10)	C18∶1		*4.0±1.0*	*5.9±0.5*	*2.6±0.5*	*4.3±0.5*		*3.6±0.5*	*8.8±0.5*	*4.3±0.5*	*6.7±0.5*
**35∶1**	**C16∶0**	**C19∶0cyc(11, 12)**	**730.54**	**17.6±0.8**	**9.1±0.5**	**13.1±0.5**	**12.0±0.5**	**761.53**	**31.6±0.8**	**13.7±0.5**	**25.7±0.5**	**27.3±0.5**
	**CH3-C16∶0**	**C18∶1**		***15.0±1.0***	***12.8±0.8***	***5.2±0.5***	***11.8±0.9***		***23.5±0.8***	***20.4±0.8***	***13.9±0.5***	***22.9±0.9***
36∶2	C18∶1	C18∶1	742.54	2.8±0.6	4.4±0.5	3.2±0.5	3.6±0.5	773.53	5.0±0.5	5.3±0.5	6.2±0.5	6.5±0.5
	C17∶0cyc(9,10)	C19∶0cyc(11, 12)		*2.7±0.6*	*5.1±0.5*	*2.3±0.5*	*3.4±0.5*		*5.0±0.5*	*10.4±0.5*	*5.2±0.5*	*7.6±0.5*
**Sum**				**93.9**	**84.8**	**86.2**	**88.5**		**74.8**	**67.2**	**71.3**	**78.6**
				***91.9***	***85.0***	***89.2***	***89.5***		***79.9***	***78.2***	***80.2***	***80.5***

Values in straight correspond to the internal membrane - *Values in italic correspond to the outer membrane.*

*Total number of C: Number of equivalent unsaturation – R and R' represent the fatty acid moiety with no indication of their position.

Note that a cyclopropylation is equivalent to an unsaturation in terms of molecular mass (e.g. the molecular masses of .C17∶0cyc and C17∶1 are identical).

In all samples, predominant PEs and PGs have a total number of from 32 to 36 carbon atoms in their aliphatic chains. However, it should be noticed that PEs and PGs with lower and higher numbers of carbon atoms were also detected.

In IM and OM enriched samples, the proportions of PEs and PGs with a total number of carbon atoms in their aliphatic chains below 32 were double in sessile bacteria (5% for PEs and 9% for PGs) compared to planktonic counterparts (2.4% for PEs and 5% for PGs). These ratios remained constant during biofilm growth. PE 30∶0 and PG 30∶0 were found in similar proportions in all samples whereas PE 30∶1 and its cyclopropylated form PE 31∶1, as well as PE 31∶0 were only observed in sessile organisms. PG 30∶1 and PG 31∶0 are present in both planktonic and sessile cells. However their proportions are double for sessile bacteria. PE 31∶0 and PG 31∶0 are constituted of one 12-CH3-C14∶0 and one C16∶0 chain. While these phospholipids were found in MS spectra from biofilm lipid extracts, their proportion did not evolve with biofilm age. The presence of these phospholipids do not correlate with the high level of 2-CH3-C14∶0 found in the fatty acid analysis from 1-day-old and 2-day-old biofilm lipid extracts. It might be explained by the presence of OLs in lipid extracts. Indeed, in 1-day-old biofilm IM enriched samples, we observed a peak corresponding to OL 31∶0 with an ester-linked 12-CH3-C14∶0 fatty acid chain. This phospholipid was absent in all other samples. However, we detected the presence of OL 32∶0 and OL 34∶1. In biofilm lipid extracts, OL 32∶0 peaks were 10 times lower in IM enriched samples and 20 times lower in OM enriched samples compared to PE 34∶1 peaks. In 1-day-old biofilm IM enriched samples, the intensity of the of OL 31∶0 peak was 3 times higher than that of OL 32∶0. The presence of these OLs in the samples is not surprising since they were already found to represent between 2 to 15% of the total *P. aeruginosa* lipids, the most abundant structures containing ester-linked C16∶0, C18∶0 and C18∶1 fatty acid chains [31]. Some ester-linked C15∶0 fatty acid chain which was likely 12-CH3-C14∶0 were also detected. Consequently, we can conclude that the presence of OL 31∶0 in the IM enriched samples at the early stage of biofilm growth may explain the high proportion of 12-CH3-C14∶0 in the fatty acid distribution. However we cannot exclude the existence of other lipids, unidentified at that time and containing such fatty acid chain.

In the case of phospholipids with a total number of carbon atoms in their aliphatic chains above 37, the proportions of PEs and PGs were respectively 6 and 3 time higher in 1-day-old biofilms lipid extracts compared to planktonic bacteria lipid extracts in both membrane samples. With the exception of PE and PG 39∶2 detected at very low levels (i.e., <1% and 3% respectively) in planktonic samples, PE and PG 38∶1, 38∶2, 39∶1 and 39∶2 as well as PG 38∶0 were detected in the 1-day-old biofilm lipid extracts. However the proportions of these phospholipids decreased with the biofilm age. These results are coherent with fatty acid analysis in which C20∶0 was clearly detected (3%) in 1-day-old and slightly detected (0.1%) in 2-day-old biofilms lipid extracts. These data show that the overall phospholipid diversity is higher in biofilms than in planktonic bacterias. The diversity decreased with the biofilm age especially for high mass phospholipids.

**Figure 2:**
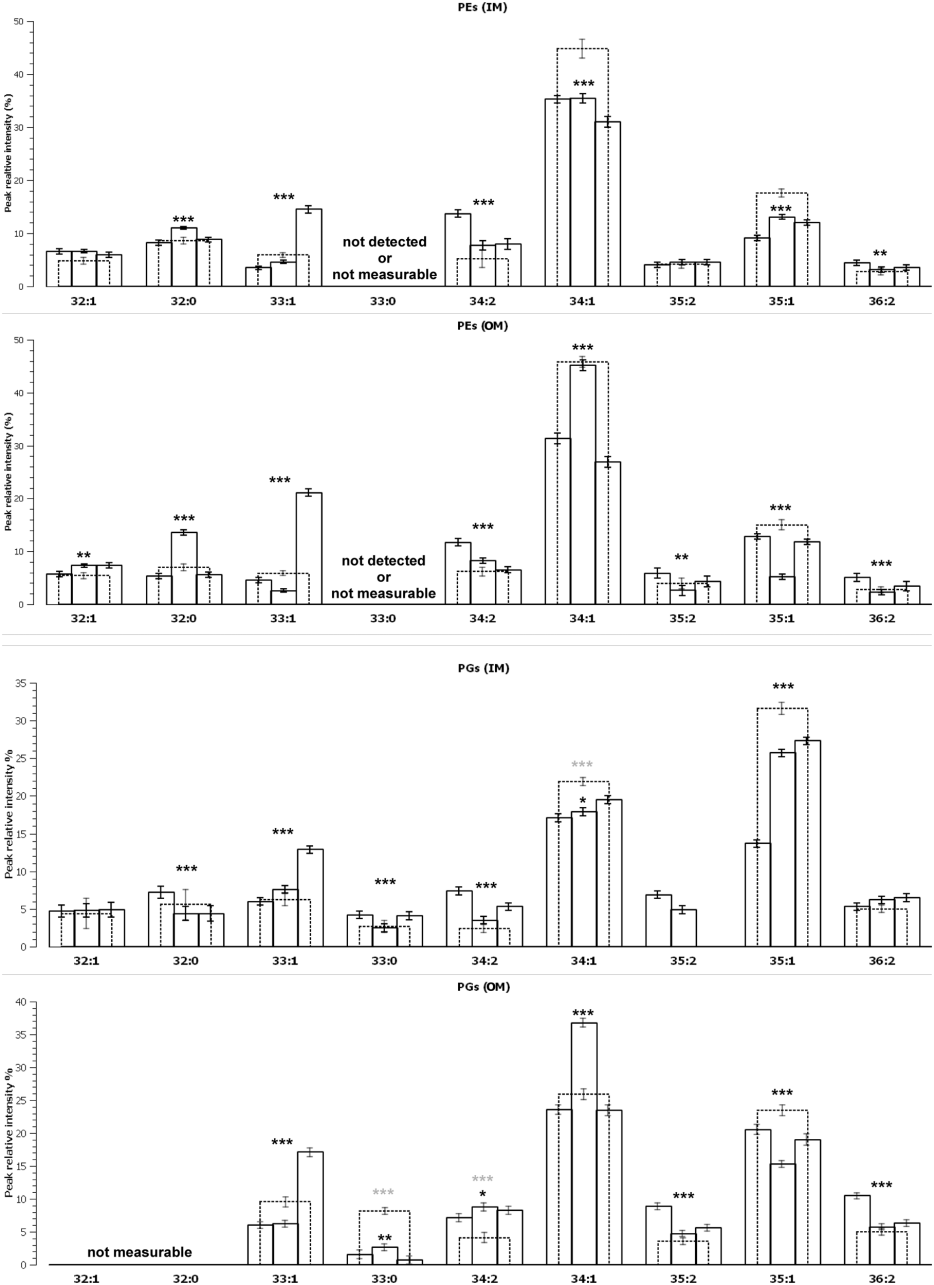
Evolution of the proportions of the main phosphatidyletnolamines and phosphatidylglycerols in *P. aeruginosa* inner and outer membranes. The proportions obtained for planktonic bacteria is represented in dashed lines. Error bars are given with a 95% confidence interval. Results of ANOVA test for the phospholipid proportions in sessile bacteria are shown (***, p-value<0,001; **, p-value<0,01; *, p-value<0,1 none p-value>0,1 (not statically relevant)). When different results of ANOVA test including the phospholipid proportions from planktonic bacteria are shown in grey.

Table 2 gives the proportions of predominant phospholipids. Uneven numbered phospholipids with one equivalent number of unsaturation (PEs and PGs 33∶1 and 35∶1) evolved differently in IM and OM enriched samples (**figure 2** **and** **table 2**). In the IM enriched samples, PE 33∶1, PG 33∶1 and PG 35∶1 increased during the biofilm formation to reach a maximum in 6-day-old sessile cells. From 1- to 6-day-old biofilm lipid extracts, the peak intensity of PE 33∶1 was increased by a factor of 4 and the peak intensity of PGs 33∶1 and 35∶1 by a factor of 2. This may be easily explained by the increase in cyclopropylated chains observed in the fatty acid analysis. However, it should be noticed that the peak intensity of PE 32∶1, PG 32∶1 and PG 34∶1 do not change with the biofilm age. This observation shows that those phospholipids are synthesised continuously during the biofilm growth. In the OM enriched samples, the evolution of PE 33∶1, PG 33∶1 and PG 35∶1 is more complex with minimum proportions in 2-day-old biofilms lipid extracts. This may be explained by the competition between the production kinetics of phospholipids with a branched chain and that of phospholipids with a cyclopropylated group. This hypothesis is reinforced by the fatty acid analysis which showed that 14-CH3-C16∶0 and 15-CH3-C16∶0 (which may be parts of PE and PG 33∶1) were abundant in 1-day-old biofilm cells and rapidly disappeared with the biofilm age. A similar remark may be done for 17-CH3-C18∶0 which may be part of PE and PG 35∶1. Conversely, C17∶0cyc(9,10) and C19∶0cyc(11,12) which may be parts of PE (or PG) 33∶1 and PE (or PG) 35∶1 respectively, increased during the biofilm formation. This may suggest that in 1-day-old biofilms, branched chains phospholipids proportions are relatively high in OM compared to IM. The proportions of these phospholipids decreased with the biofilm age. In addition, like in IM, cyclopropylated chains phospholipids increased continuously during the biofilm formation. The combination of these alterations explains the evolution of PEs and PGs 33∶1 and 35∶1 in OM enriched samples.

In 2-day-old biofilm OM enriched samples, the proportions of PE 34∶1 and PG 34∶1 exhibited an optimum value, while the proportions of PE 35∶1 and PG 35∶1 exhibited a minimum value. We also observed that in most cases even numbered phospholipids bearing 2 unsaturations continuously decreased during the biofilm growth. All these results show an obvious diminution of unsaturated phospholipids during the biofilm growth. This observation disagrees with the fatty acid analysis but accords well with Chao *et al* results [28].

## Conclusion

Compared with that of planktonic organisms, the lipid distribution in biofilm bacterial membranes exhibited alterations in terms of acyl chains length, nature of the phosphate substituent and nature of the polar head group of glycerophospholipids (PG and PE), confirming previous observations [19]. An outstanding information brought by the present study is that these lipidome alterations are biofilm age dependent, changes being higher in the early phase of the biofilm formation. It has been shown that the diversity of glycerophospholipids within the membrane is the result of numerous combinations of acyl chains and head groups and is crucial to maintain the bilayer structure, the cell permeability and the membrane fluidity [32]. In conclusion, the alterations of the IM and OM lipid compositions which we observed here reflect a bacterial adaptation to the environment conditions prevailing in biofilm.

In mature biofilms (6-day-old), the phospholipid distribution seemsclose to that of planktonic bacteria with some notable exception, however, e.g. PE and PG 33∶1 (C17∶0cyc(9,10) and C16∶0 fatty acid chains) which are more abundant in 6-day-old biofilms. This observation might suggest a progressive “bacteria conditioning” to a return to the planktonic mode.

## References

[1] Petrova OE, Sauer K (2009) A novel signaling network essential for regulating *Pseudomonas aeruginosa* biofilm development, PLoS Pathog. 5(11): e1000668. 10.1371/journal.ppat.1000668 PMC2774163 19936057

[2] Tummler B, Koopmann U, Grothues D, Weissbrodt H, Steinkamp G, et al (1991) Nosocomial acquisition of Pseudomonas aeruginosa by cystic fibrosis patients. J Clin Microbiol. 29: 1265–1267. 10.1128/jcm.29.6.1265-1267.1991 PMC271975 1907611

[3] Lambert PA (2002) Mechanisms of antibiotic resistance in *Pseudomonas aeruginosa.* . J R Soc Med 95: 22–26. 12216271 PMC1308633

[4] Strateva T, Yordanov D (2009) *Pseudomonas aeruginosa* – a phenomenon of bacterial resistance. J Med Microbiol. 58: 1133–1148. 10.1099/jmm.0.009142-0 19528173

[5] Costerton JW, Stewart PS, Greenberg EP (1999) Bacterial biofilms: a common cause of persistent infections. Science. 284: 1318–1322. 10.1126/science.284.5418.1318 10334980

[6] Mah TF, O'Toole GA (2001) Mechanisms of biofilm resistance to antimicrobial agents. Trends Microbiol. 9: 34–39. 10.1016/s0966-842x(00)01913-2 11166241

[7] Schierholz JM, Beuth J, Pulverer G (1999) Adherent bacteria and activity of antibiotics, J. Antimicrob. Chemother. 43: 158–160. 10.1093/jac/43.1.158 10381118

[8] Drenkard E (2003) Antimicrobial resistance of *Pseudomonas aeruginosa* biofilms. Microbes Infect. 5: 1213–1219. 10.1016/j.micinf.2003.08.009 14623017

[9] Stewart PS (2002) Mechanisms of antibiotic resistance in bacterial biofilms. Int J Med Microbiol. 292: 107–113. 10.1078/1438-4221-00196 12195733

[10] Whiteley M, Bangera MG, Bumgarnes RE, Parsek MR, Teitzeil GM, et al (2001) Gene expression in *Pseudomonas aeruginosa* biofilms. Nature 413: 860–864. 11677611 10.1038/35101627

[11] Schembri MA, Kjærgaard K, Klemm P (2001) Global gene expression in *Escherichia coli* biofilms. Mol. Microbiol. 48: 253–267. 10.1046/j.1365-2958.2003.03432.x 12657059

[12] Vilain S, Cosette P, Hubert M, Lange C, Junter GA, et al (2004) Comparative proteomic analysis of planktonic and immobilized *Pseudomonas aeruginosa* cells: a multivariate statistical approach. Anal Biochem 329: 120–130. 15136174 10.1016/j.ab.2004.02.014

[13] Junter GA, Jouenne T (2004) Immobilized viable microbial cells: from the process to the proteome em leader or the cart before the horse. Biotechnol Adv. 22: 633–658. 10.1016/j.biotechadv.2004.06.003 15364350

[14] Folstom JP, Richards L, Pitts B, Roe F, Ehrlich GD, et al. (2010) Physiology of *Pseudomonas aeruginosa* in biofilms as revealed by transcriptome analysis. BMC Microbiology, 10∶294. doi: 10.1186/1471-2180-10-294. 10.1186/1471-2180-10-294 PMC2998477 21083928

[15] Zhang B, Powers R (2012) Analysis of bacterial biofilm using NMR-based metabolomics. Future Med. Chem. 4: 1273–1306. 10.4155/fmc.12.59 PMC3564560 22800371

[16] Denich TJ, Beaudette LA, Lee H, Trevors JT (2003) Effect of selected environmental and physico-chemical factors on bacterial cytoplasmic membranes. J. Microbiol. Methods. 52: 149–182. 10.1016/s0167-7012(02)00155-0 12459238

[17] O’Toole GA, Pratt LA, Watnick PI, Newman DK, Weaver VB, et al (1999) Genetic approaches to study of biofilms. Methods Enzymol. 310: 91–109. 10.1016/s0076-6879(99)10008-9 10547784

[18] Abdul Lattif A, Mukherjee PK, Chandra J, Roth MR, Welti R, et al (2011) Lipidomics of *Candida albicans* biofilms reveals phase-dependent production of phospholipid molecular classes and role for lipid rafts in biofilm formation. Microbiology. 157: 3232–3242. 10.1099/mic.0.051086-0 PMC3352276 21903752

[19] Benamara H, Rihouey C, Jouenne T, Alexandre S (2011) Impact of the biofilm mode of growth on the inner membrane phospholipid composition and lipid domains in *Pseudomonas aeruginosa*. Biochim Biophys Acta. 1808: 98–105. 10.1016/j.bbamem.2010.09.004 20849811

[20] Mizuno T, Kageyama M (1978) Separation and characterization of the outer membrane of *Pseudomonas aeruginosa*, J Biochem. 84: 179–191. 10.1093/oxfordjournals.jbchem.a132106 99443

[21] Winsor GL, Lam DK, Fleming L, Lo R, Whiteside MD, et al.. (2011). Pseudomonas Genome Database: improved comparative analysis and population genomics capability for Pseudomonas genomes. Nucleic Acids Res. 39(Database issue): D596–600. 10.1093/nar/gkq869 PMC3013766 20929876

[22] Osborn MJ, Gander JE, Parisi E, Carson J (1972) Mechanism of assembly of the outer membrane of *Salmonella typhimurium*: Isolation and characterization of cytoplasmic and outer membrane, J. Biol. Chem. 247: 3962–3972. 4555955

[23] Bligh EG, Dyer WJ (1959) A rapid method for total lipid extraction and purification. Can J Biochem Physiol. 37: 911–917. 10.1139/o59-099 13671378

[24] Raheja RK, Kaur C, Singh A, Bhatia IS (1973) New colorimetric method for the quantitative estimation of phospholipids without acid digestion. J Lipid Res. 14: 695–697. 4742564

[25] Steger K, Jarvis A, Smars S, Sundh I (2003) Comparison of signature lipid methods to determine microbial community structure in compost. J Microbiol Methods. 55: 371–382. 10.1016/s0167-7012(03)00187-8 14529958

[26] Hoffmann M, Wagner M, Abbadi A, Fulda M, Feussner I (2008) Metabolic engineering of_3-very long chain polyunsaturated fatty acid production by an exclusively acyl-CoA-dependent pathway. J. Biol. Chem. 283: 22352–22362. 10.1074/jbc.M802377200 18556660

[27] Vilain S, Cosette P, Zimmerlin I, Dupont JP, Junter GA, et al (2004) Biofilm proteome: homogeneity or versatility? J Proteome Res. 3(1): 132–6. 10.1021/pr034044t 14998174

[28] Chao J, Wolfaardt GM, Arts MT (2011) Characterization of *Pseudomonas aeruginosa* fatty acid profiles in biofilms and planktonic cultures. Can. J. Microbiol. 56: 1028–1039. 10.1139/W10-093 21164573

[29] Milne S, Ivanova P, Forrester J, Alex Brown H (2006) Lipidomics: an analysis of cellular lipids by ESI-MS. Methods 39(2): 92–103. 16846739 10.1016/j.ymeth.2006.05.014

[30] Vasseur P, Soscia C, Voulhoux R, Filloux A (2007) PelC is a *Pseudomonas aeruginosa* outer membrane lipoprotein of the OMA family of proteins involved in exopolysaccharide transport. Biochimie 89: 903–915. 17524545 10.1016/j.biochi.2007.04.002

[31] Kawai Y, Yano I, Kaneda K, Yabuuch E (1988) Ornithine-containing lipids of some *Pseudomonas* species. Eur. J. Biochem. 175: 633–641. 10.1111/j.1432-1033.1988.tb14239.x 3409887

[32] Dowhan W (1997) Molecular basis for membrane phospholipid diversity: why are there so many lipids? Annu Rev Biochem. 66: 199–232. 10.1146/annurev.biochem.66.1.199 9242906

